# Association of Mental Health Disorders With Health Care Utilization and Costs Among Adults With Chronic Disease

**DOI:** 10.1001/jamanetworkopen.2019.9910

**Published:** 2019-08-23

**Authors:** Barbora Sporinova, Braden Manns, Marcello Tonelli, Brenda Hemmelgarn, Frank MacMaster, Nicholas Mitchell, Flora Au, Zhihai Ma, Robert Weaver, Amity Quinn

**Affiliations:** 1 Department of Medicine, Cumming School of Medicine, University of Calgary, Calgary, Alberta, Canada; 2 Alberta Health Services, Edmonton, Alberta, Canada; 3 Libin Cardiovascular Institute, O’Brien Institute of Public Health, Cumming School of Medicine, University of Calgary, Calgary, Alberta, Canada; 4 Department of Community Health Sciences, Cumming School of Medicine, University of Calgary, Calgary, Alberta, Canada; 5 Department of Psychiatry, Cumming School of Medicine, University of Calgary, Calgary, Alberta, Canada; 6 Strategic Clinical Network for Addictions and Mental Health, Alberta Health Services, Edmonton, Alberta, Canada; 7 Department of Psychiatry, University of Alberta, Edmonton, Alberta, Canada

## Abstract

**Importance:**

A population-based study using validated algorithms to estimate the costs of treating people with chronic disease with and without mental health disorders is needed.

**Objective:**

To determine the association of mental health disorders with health care costs among people with chronic diseases.

**Design, Setting, and Participants:**

This population-based cohort study in the Canadian province of Alberta collected data from April 1, 2012, to March 31, 2015, among 991 445 adults 18 years and older with a chronic disease (ie, asthma, congestive heart failure, myocardial infarction, diabetes, epilepsy, hypertension, chronic pulmonary disease, or chronic kidney disease). Data analysis was conducted from October 2017 to August 2018.

**Exposures:**

Mental health disorder (ie, depression, schizophrenia, alcohol use disorder, or drug use disorder).

**Main Outcomes and Measures:**

Resource use, mean total unadjusted and adjusted 3-year health care costs, and mean total unadjusted 3-year costs for hospitalization and emergency department visits for ambulatory care–sensitive conditions.

**Results:**

Among 991 445 participants, 156 296 (15.8%) had a mental health disorder. Those with no mental health disorder were older (mean [SD] age, 58.1 [17.6] years vs 55.4 [17.0] years; *P* < .001) and less likely to be women (50.4% [95% CI, 50.3%-50.5%] vs 57.7% [95% CI, 57.4%-58.0%]; *P* < .001) than those with mental health disorders. For those with a mental health disorder, mean total 3-year adjusted costs were $38 250 (95% CI, $36 476-$39 935), and for those without a mental health disorder, mean total 3-year adjusted costs were $22 280 (95% CI, $21 780-$22 760). Having a mental health disorder was associated with significantly higher resource use, including hospitalization and emergency department visit rates, length of stay, and hospitalization for ambulatory care–sensitive conditions. Higher resource use by patients with mental health disorders was not associated with health care presentations owing to chronic diseases compared with patients without a mental health disorder (chronic disease hospitalization rate per 1000 patient days, 0.11 [95% CI, 0.11-0.12] vs 0.06 [95% CI, 0.06-0.06]; *P* < .001; overall hospitalization rate per 1000 patient days, 0.88 [95% CI, 0.87-0.88] vs 0.43 [95% CI, 0.43-0.43]; *P* < .001).

**Conclusions and Relevance:**

This study suggests that mental health disorders are associated with substantially higher resource utilization and health care costs among patients with chronic diseases. These findings have clinical and health policy implications.

## Key Points

**Question:**

Are mental health disorders associated with health care utilization and costs among people with chronic diseases?

**Findings:**

In this population-based cohort study of 991 445 Canadian adults, including 156 296 with a mental health disorder, 3-year adjusted mean costs were $38 250 for those with a mental health disorder and $22 280 for those without a mental health disorder. Presence of a mental health disorder was associated with higher rates of hospitalization and emergency department visits, including when considering only visits associated with chronic disease and ambulatory care–sensitive conditions.

**Meaning:**

In this study, mental health disorders were associated with substantially higher resource utilization and health care costs in patients with chronic diseases.

## Teaser

This population-based cohort study determines the association of mental health disorders with health care utilization and costs for Canadian adults with chronic disease.

## Introduction

Chronic diseases, such as diabetes, heart disease, and chronic kidney disease, are common, represent a significant burden for patients and payers,^1^ and are projected to constitute 60% of global disease burden by 2020.^2^ Mental health and substance use disorders also contribute significantly to the global burden of disease. In 2010, mental and substance use disorders represented 7.4% of total disease burden worldwide, were responsible for more of the global burden than HIV/AIDS, tuberculosis, or diabetes, and were the leading global cause of all nonfatal burden of disease.^3^

There is a recognized association between mental and physical health. For example, mortality in cancer, diabetes, and following a heart attack is higher for patients with depression.^4,5^ Further, compared with the general population, people with chronic disease have higher rates of mental health disorders, while people with mental health disorders have a greater risk of developing chronic diseases.^6,7,8,9^

Canada’s provincial health care spending has more than doubled in the past 15 years.^10^ Both mental health disorders and chronic diseases have a substantial impact on health care costs, leading to significant economic loss and disability.^5^ The cost of managing patients with chronic diseases represents around 60% of Canada’s $228 billion annual health care budget.^11^ A 2003 study^12^ estimated the direct and indirect burden of mental health disorders to the Canadian economy at $51 billion, with similar estimates published in 2011.^13^ A 2016 study estimated the impact of only depression on the economy at $32.3 billion.^14^ In addition to the high costs, mental health and chronic disease outcomes for patients with mental health disorders are poor,^5,15,16^ suggesting the need for a closer examination of care delivery to patients with mental health disorders.

A few studies^17,18,19,20,21^ have examined the association of having a mental health disorder with the cost of care for individuals with other chronic diseases, but they generally have been small, have not always used validated algorithms to define mental health disorders, and have usually focused on a single chronic disease. Further, no Canadian estimates are available. Given this, we sought to determine the association of having a mental health disorder with health care utilization and costs for patients with chronic diseases.

## Methods

### Cohort

Our cohort included all adults 18 years and older in Alberta, Canada, with at least 1 of the following chronic diseases as of April 1, 2012: asthma, congestive heart failure, myocardial infarction, diabetes, epilepsy, hypertension, chronic pulmonary disease, and chronic kidney disease. These diseases were selected because they are common and often coexist, and a list of ambulatory care–sensitive conditions (ACSCs) for these conditions has been defined by the Canadian Institute of Health Information.^22^ Good-quality outpatient care would be expected to lower the risk of hospitalization and emergency department (ED) visits for ACSCs.^22^ We included chronic kidney disease because these patients share similar risk factors, and a list of ACSCs is also available for this group.^23^ Patients with chronic disease as of April 1, 2012, were defined based on hospitalization and physician billing claims from April 1, 1994, to April 1, 2012, using validated algorithms based on *International Classification of Diseases, Ninth Revision* [*ICD*-*9*] and *ICD*-*10* codes^24^ (eTable 1 in the Supplement). We defined chronic kidney disease based on laboratory measures as in our previous work^25^ and consistent with international guidelines^26^ (eTable 1 in the Supplement). Patients had 3 years of follow up after the index date of April 1, 2012, with censoring for death and movement out of Alberta.

### Data

We used the Interdisciplinary Chronic Disease Collaboration Data Repository, described previously,^27,28,29,30^ which includes vital statistics, prescription drug data, physician claims, hospitalizations, ED and outpatient visits, laboratory data, and health care costs for all Alberta residents enrolled in Alberta Health coverage from April 1, 1994, to March 31, 2015. Of note, more than 99% of Alberta residents participate in the public health insurance offered by the government-sponsored Alberta Health. Ethical approval for this study was obtained from the University of Calgary Health Ethics Research Board. Informed consent was waived owing to the use of deidentified data. This study followed the Strengthening the Reporting of Observational Studies in Epidemiology (STROBE) reporting guideline.

### Variables

The explanatory variable was having a mental health disorder (ie, depression, schizophrenia, or substance use disorder, including alcohol or drug use disorders) from April 1, 1994, to April 1, 2012. These mental health disorders were selected because of their importance but also because they can be identified with high- or moderate-validity diagnostic algorithms (high-validity diagnostic algorithms: positive predictive value and sensitivity, ≥70%; moderate-validity diagnostic algorithms: positive predictive value, ≥70%; sensitivity, <70%) using health administrative data^24,31^ (eTable 2 in the Supplement). The timing of the mental health disorder diagnosis in relation to the chronic disease diagnosis was not assessed.

Additional covariates included age, sex, rural residence, 26 comorbidities (listed in Table 1 footnote),^24^ neighborhood-income level (an estimate of socioeconomic status), and resource use. Neighborhood-level income was defined as annual adjusted median neighborhood-level household income quintiles, estimated by linking patient postal code to census data.

**Table 1.** Baseline Demographic Characteristics for Adults With Chronic Disease, With and Without a Mental Health Disorder, in Alberta, 2012

Variable	% (95% CI)					
	No Mental Health Disorder Coded (n = 835 149)	Mental Health Disorder Coded (n = 156 296)	Depression (n = 110 671)	Schizophrenia (n = 13 320)	Alcohol Use Disorder (n = 40 955)	Drug Use Disorder (n = 31 465)
Age, mean (SD), y	58.1 (17.6)	55.4 (17.0)^a^	55.9 (17.2)	53.4 (16.4)	53.7 (16.0)	49.3 (15.5)
Women	50.4 (50.3-50.5)	57.7 (57.4-58.0)^a^	66.1 (65.7-66.3)	50.0 (49.1-50.8)	35.3 (34.8-35.7)	48.8 (48.2-49.3)
Neighborhood income^b^						
High	16.7 (16.6-16.7)	12.8 (12.6-12.9)^a^	13.9 (13.7-14.1)	9.0 (8.6-9.5)	9.6 (9.3-9.9)	9.3 (9.0-9.7)
Low	25.1 (25.0-25.2)	30.2 (30.0-30.4)^a^	28.3 (28.1-28.6)	39.2 (38.4-40.1)	35.5 (35.0-35.9)	37.1 (36.6-37.6)
Rural residence	14.1 (14.0-14.2)	13.5 (13.4-13.7)^a^	11.8 (11.6-12.0)	9.7 (9.2-10.2)	18.5 (18.1-18.9)	15.4 (15.0-15.8)
Chronic disease						
Asthma	7.1 (7.0-7.1)	12.6 (12.5-12.8)^a^	12.2 (12.0-12.4)	17.0 (16.4-17.7)	14.5 (14.2-14.9)	20.0 (19.6-20.5)
Congestive heart failure	8.4 (8.3-8.5)	11.9 (11.8-12.1)^a^	11.3 (11.1-11.5)	11.0 (10.5-11.5)	13.7 (13.4-14.0)	11.4 (11.1-11.8)
Myocardial infarction	4.3 (4.2-4.3)	4.5 (4.4-4.6)^a^	3.9 (3.7-4.0)	3.2 (2.9-3.5)	5.6 (5.4-5.9)	4.4 (4.2-4.7)
Diabetes	24.3 (24.2-24.4)	24.5 (24.2-24.7)	24.1 (23.8-24.3)	31.8 (31.0-32.6)	24.7 (24.3-25.1)	21.8 (21.3-22.2)
Epilepsy	3.7 (3.7-3.8)	9.0 (8.9-9.2)^a^	7.5 (7.4-7.7)	15.5 (14.8-16.1)	14.5 (14.2-14.9)	15.9 (15.5-16.3)
Hypertension	67.5 (67.4-67.6)	61.4 (61.1-61.6)^a^	63.5 (63.2-63.8)	53.0 (52.1-53.8)	57.3 (56.8-57.8)	48.2 (47.6-48.7)
Chronic pulmonary disease	22.8 (22.7-22.9)	33.3 (33.1-33.6)^a^	31.0 (30.7-31.3)	34.6 (33.8-35.4)	40.9 (40.4-41.4)	42.3 (41.7-42.8)
Chronic kidney disease	23.3 (23.2-23.4)	24.3 (24.1-24.5)^a^	25.6 (25.3-25.8)	23.1 (22.4-23.8)	20.6 (20.2-21.0)	19.3 (18.8-19.7)
No. of comorbidities^c^						
1	37.5 (37.3-37.6)	25.5 (25.3-25.7)^a^	25.9 (25.6-26.2)	23.6 (22.8-24.3)	22.6 (22.2-23.0)	23.2 (22.7-23.7)
2-3	45.2 (45.0-45.3)	44.4 (44.1-44.6)^a^	44.3 (44.0-44.5)	43.0 (42.1-43.8)	43.8 (43.3-44.2)	44.1 (43.5-44.7)
4-6	15.2 (15.1-15.3)	24.2 (24.0-24.4)^a^	23.8 (23.6-24.1)	26.6 (25.8-27.3)	27.1 (26.7-27.5)	25.8 (25.3-26.3)
>6	2.2 (2.2-2.3)	6.0 (5.9-6.1)^a^	6.0 (5.9-6.1)	6.9 (6.5-7.3)	6.5 (6.3-6.8)	6.9 (6.6-7.2)

^a^ Denotes *P* < .001 for comparison between mental health disorder absent and mental health disorder present.

^b^ Income categories were estimated using postal codes. High income represents the highest quintile, and low income represents the lowest quintile.

^c^ Comorbidities include asthma, atrial fibrillationchronic hepatitis B, chronic kidney disease, chronic pain, chronic pulmonary disease, cirrhosis, congestive heart failure, dementia, diabetes, epilepsy, hypertension, hypothyroidism, inflammatory bowel disease, irritable bowel syndrome, lymphoma, metastatic cancer, multiple sclerosis, myocardial infarction, nonmetastatic cancer, Parkinson disease, peptic ulcer disease, peripheral vascular disease, psoriasis, rheumatoid arthritis, and stroke or transient ischemic attack.

### Primary Outcome

The primary outcome was mean total 3-year health care costs for patients with and without a mental health disorder. We included costs for hospitalization and ED visits, physician claims (specialist and primary care physician visit and procedure billing costs), prescription medications, nonphysician ambulatory costs (day medicine and day surgery clinics), and outpatient diagnostic imaging and laboratory costs. The total costs were calculated as the sum of these costs. Alberta Health uses Canadian Institute of Health Information case-mix grouper methods to estimate hospital costs and ambulatory-case costing methods to estimate outpatient costs.^32,33^ Physician claims were based on the amount paid by Alberta Health. Reason for hospitalization was deemed associated with a mental health disorder or a chronic disease if the most responsible diagnosis was an *ICD*-*9* or *ICD*-*10* code associated with the mental health disorder or the chronic disease of interest. The cost of medications was estimated by combining a database containing a comprehensive list of medications dispensed to all Alberta residents with a price list from Alberta Blue Cross, including dispensing fee. Diagnostic imaging and laboratory costs were based on estimates provided by Alberta Health Services. All costs are reported in 2016 Canadian dollars.^34^

### Secondary Outcomes

The secondary outcomes, defined for the 3 years assessed, were resource use and costs of hospitalizations and ED visits for ACSCs for the same index chronic diseases^22,23^ in people with and without mental health disorders (eTable 3 in the Supplement). We defined 3 measures to characterize participants’ health care resource use as follows: (1) unadjusted rate per 1000 patient days of all-cause and chronic disease–specific hospitalizations and ED visits, (2) mean total all-cause and chronic disease–specific hospital length of stay, and (3) rates of hospitalizations and ED visits for ACSCs. Hospitalization and ED visit causes were defined as the most responsible *ICD*-*10* code.

### Statistical Analysis

All analyses were performed for those without mental health disorders, those with any mental health disorder, and for each of the 4 mental health disorders. The unadjusted mean 3-year costs were further characterized by costing category. Differences in demographic characteristics, resource use, and unadjusted costs between those with and without mental health disorders were determined with *t* tests and *z* tests for continuous variables and χ^2^ tests for categorical variables. Differences in unadjusted costs between the 4 mental health disorders were determined with a factorial analysis of variance. Differences were considered significant at *P* < .05, and all tests were 2-tailed.

To test for an association of type of mental health disorder with costs, we used multivariable linear regression to predict total costs, controlling for age, sex, socioeconomic status, rural status, medical comorbidities, and mental health disorders. We then used β coefficients to estimate mean total adjusted costs for each mental health disorder.

Before conducting regression modeling, we examined the cost distribution. While we did not identify problems with 0-costs and censoring, the distribution of the total costs was positively skewed, while the log of the total costs was more normally distributed. To identify the model with the best fit for our data, we first conducted a modified Park test to determine the appropriate family of the model,^35^ which indicated the γ distribution more appropriately fit our regression than the Poisson distribution. We then compared the fit of a linear regression model using ordinary least squares estimation against 5 other models (ie, 1 linear regression of log total costs with smearing transformation; 3 generalized linear models of log total costs using the negative binomial, γ, and inverse Gaussian distributions; and 1 generalized linear model of total costs using the inverse Gaussian distribution). The ordinary least squares model had the best fit based on the root-mean-square error (lowest of all the models) and pseudo-*R*^2^ (highest of all the models). Given the ease of interpretation and model fit, we present ordinary least squares results. Analyses were completed using Stata version 14 (StataCorp).

## Results

### Baseline Characteristics

Between April 1, 1994, and March 31, 2012, 991 445 adults with a chronic disease were identified; 156 296 (15.8%) had a concomitant mental health disorder (Table 1). Depression was the most common mental health disorder, present in 110 671 (11.2%) individuals in the cohort. In those with depression, 10.3% had a concomitant substance use disorder (eFigure in the Supplement). People with a mental health disorder were more likely to be younger (mean [SD] age, 55.4 [17.0] years vs 58.1 [17.6] years of age; *P* < .001), female (57.7% [95% CI, 57.4%-58.0%] vs 50.4% [95% CI, 50.3%-50.5%]; *P* < .001), be of lower socioeconomic status (30.2% [95% CI, 30.0%-30.4%] vs 25.1% [95% CI, 25.0%-25.2%]; *P* < .001), have 4 or more comorbidities (4-6 comorbidities: 24.2% [95% CI, 24.0%-24.4%] vs 15.2% [95% CI, 15.1%-15.3%]; *P* < .001; >6 comorbidites: 6.0% [95% CI, 5.9%-6.1%] vs 2.2% [95% CI, 2.2%-2.3%]; *P* < .001), and were more likely to die during the 3-year study period (8.9% [95% CI, 8.4%-9.4%] vs 4.9% [95% CI, 4.7%-5.1%]; *P* < .001; not shown in Table 1).

### Mean Total 3-Year Costs

The mean total 3-year unadjusted costs of patients with chronic disease were $20 210 (95% CI, $19 674-$20 750) for those without a mental health disorder and $38 250 (95% CI, $36 476-$39 935) for those with a mental health disorder. For patients with a chronic disease and depression, total 3-year unadjusted costs were $34 690 (95% CI, $33 580-$35 810), for patients with a chronic disease and schizophrenia, $50 450 (95% CI, $47 460-$53 440), for patients with a chronic disease and alcohol use disorder, $42 320 (95% CI, $40 030-$44 620), and for patients with a chronic disease and drug use disorder, $45 260 (95% CI, $42 590-$47 920) (*P* < .001, for all estimates) (Table 2). The highest cost categories for all groups were hospitalizations, prescription drugs, and physician visits.

**Table 2.** Mean Total 3-Year Unadjusted Costs for Adults With Chronic Disease, With and Without a Mental Health Disorder

Cost	Mean Cost (95% CI), Can$^a^					
	No Mental Health Disorder Coded (n = 835 149)	Mental Health Disorder Coded (n = 156 296)	Depression (n = 110 671)	Schizophrenia (n = 13 320)	Alcohol Use Disorder (n = 40 955)	Drug Use Disorder (n = 31 465)
Hospitalization	6610 (6650-6660)	14 250 (14 030-14 470)	13 720 (13 470-13 960)	23 840 (22 838-24 840)	19 950 (19 420-20 470)	19 340 (18 760-19 930)
Physician visit	3360 (3350-3370)	6080 (6040-6120)	6320 (6270-6360)	9790 (9560-10 010)	6700 (6610-6790)	7490 (7370-7600)
Prescription	6090 (5560-6620)	8180 (7200-9150)	8110 (7040-9170)	9370 (6680-12 060)	8310 (6140-10 490)	10 580 (8400-13 100)
Emergency department	850 (850-860)	1820 (1800-1840)	1750 (1730-1780)	2180 (2110-2260)	2640 (2590-2690)	2940 (2880-3000)
Nonphysician ambulatory care^b^	2020 (2000-2040)	3060 (3000-3120)	3000 (3010-3150)	3980 (3712-4250)	3120 (2990-3250)	3360 (3190-3530)
Diagnostic imaging^c^	980 (980-990)	1230 (1220-1240)	1310 (1300-1320)	830 (800-850)	1090 (1080-1110)	1090 (1080-1120)
Laboratory test	290 (290-290)	410 (410-420)	400 (400-410)	460 (450-480)	510 (500-520)	470 (460-470)
Total cost	20 210 (19 674-20 750)	35 030 (34 010-36 050)^d^	34 690 (33 580-35 810)	50 450 (47 460-53 440)	42 320 (40 030-44 620)	45 260 (42 590-47 920)

^a^ All cost estimates are adjusted to 2016 Canadian dollars.

^b^ Day medicine and day surgery.

^c^ Outpatient diagnostic imaging.

^d^ Denotes *P* < .001 for comparison between mental health disorder absent and mental health disorder present.

When we examined differences in costs by individual mental health disorders and age, sex, income, and rural status, higher cost was associated with the presence of a mental health disorder, being older than 65 years, and low income (eTable 4 in the Supplement). Costs for older people with co-occurring depression and substance use disorders were very high ($74 119; 95% CI, $65 415-$82 823 vs older adults without a mental health disorder: $29 401; 95% CI, $28 577-$30 227; *P* < .001) (eTable 4 in the Supplement).

Furthermore, when we examined costs by individual mental health disorders and number of comorbidities, we found costs were higher for patients with any mental health disorder and with increasing number of comorbidities (eTable 5 in the Supplement). Total costs were significantly higher for patients with 3 or more comorbidities. Compared with those without a mental health diagnosis in all adults with fewer than 7 comorbidities, adults with depression had significantly higher costs ($32 069; 95% CI, $30 826-$33 311 vs $38 102; 95% CI, $36 497-$39 708; *P* < .001) (eTable 5 in the Supplement).

### Adjusted Costs: Multivariable Regression Analysis

The results from the regression model are presented in eTable 6 in the Supplement. The adjusted mean total 3-year costs for patients without a mental health disorder were $22 280 (95% CI, $21 780-$22 760; *P* < .001); for those with depression, $37 990 (95% CI, $36 250-$39 720; *P* < .001); for those with schizophrenia, $47 740 (95% CI, $43 380-$52 100; *P* < .001); for those with alcohol use disorder, $38 300 (95% CI, $35 550-$41 040; *P* < .001); and for those with drug use disorder, $42 440 (95% CI, $39 350-$45 530; *P* < .001) (Figure).

**Figure.**
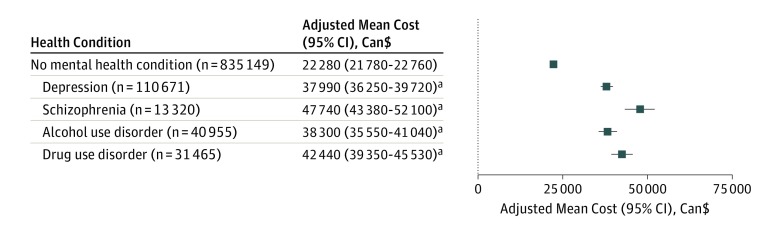
Adjusted Mean 3-Year Cost per Patient for Each Mental Health Disorder Costs are adjusted for sex, age, socioeconomic status, comorbidities (ie, asthma, atrial fibrillationchronic hepatitis B, chronic kidney disease, chronic pain, chronic pulmonary disease, cirrhosis, congestive heart failure, dementia, diabetes, epilepsy, hypertension, hypothyroidism, inflammatory bowel disease, irritable bowel syndrome, lymphoma, metastatic cancer, multiple sclerosis, myocardial infarction, nonmetastatic cancer, Parkinson disease, peptic ulcer disease, peripheral vascular disease, psoriasis, rheumatoid arthritis, and stroke or transient ischemic attack), and mental health disorders (ie, depression, schizophrenia, alcohol use disorder, and drug use disorder). Adjustment was performed using ordinary least squares linear regression and log transformations of costs were not performed. All cost estimates are adjusted to 2016 Canadian dollars. ^a^Denotes *P* < .001 for comparison between mental health disorder absent and mental health disorder present.

### Resource Use

Although 15.8% of our cohort (156 296 individuals) had a mental health diagnosis, patients with mental health disorders accounted for 140 560 of 522 140 admissions (26.9%). Of their admissions, only approximately 16 029 (11.4%) were for the care of 1 of the 4 mental health disorders (data not shown). The reasons for admission were broad, including infectious causes, surgical presentations, and gastrointestinal issues (data not shown). Resource use by patients with mental health disorders was not driven by health care presentations owing to chronic diseases compared with patients without a mental health disorder (rate of hospitalization for chronic disease per 1000 patient days: 0.06 [95% CI, 0.06-0.06] vs 0.11 [95% CI, 0.11-0.12]; *P* < .001; overall rate of hospitalization per 1000 patient days: 0.43 [95% CI, 0.43-0.43] vs 0.88 [95% CI, 0.87-0.88]; *P* < .001) (Table 3).

**Table 3.** Resource Use for Adults With Chronic Disease, With and Without a Mental Health Disorder

Resource	Rate (95% CI)					
	No Mental Health Disorder Coded (n = 835 149)	Mental Health Disorder Coded (n = 156 296)	Depression (n = 110 671)	Schizophrenia (n = 13 320)	Alcohol Use Disorder (n = 40 955)	Drug Use Disorder (n = 31 465)
**All-Cause Hospitalizations or ED Visits**						
Hospitalizations per 1000 patient-days	0.43 (0.43-0.43)	0.88 (0.87-0.88)^a^	0.85 (0.84-0.86)	1.17 (1.13-1.20)	1.25 (1.23-1.27)	1.24 (1.22-1.27)
Mean total hospital LOS over 3 y, d	4.7 (4.6-4.7)	11.6 (11.4-11.8)^b^	11.5 (11.2-11.7)	24.8 (23.5-26.2)	15.6 (15.0-16.1)	15.9 (15.2-16.5)
ED visits per 1000 patient-days	1.75 (1.74-1.76)	3.75 (3.72-3.79)^a^	3.57 (3.53-3.62)	4.45 (4.29-4.61)	5.48 (5.38-5.57)	6.35 (6.21-6.49)
**Chronic Disease–Associated Hospitalizations or ED Visits**^c^						
Hospitalizations for chronic disease per 1000 patient-days	0.06 (0.06-0.06)	0.11 (0.11-0.12)^a^	0.10 (0.10-0.11)	0.11 (0.10-0.12)	0.17 (0.15-0.18)	0.16 (0.15-0.17)
Mean total hospital LOS for chronic disease over 3 y, d	0.6 (0.6-0.6)	1.3 (1.2-1.4)^b^	1.2 (1.2-1.3)	1.5 (1.3-1.7)	1.6 (1.5-1.8)	1.9 (1.8-2.0)
ED visits for chronic disease per 1000 patient-days	0.13 (0.13-0.14)	0.27 (0.26-0.28)^a^	0.24 (0.23-0.25)	0.28 (0.24-0.31)	0.42 (0.39-0.44)	0.43 (0.40-0.46)
**Hospitalizations or ED Visits for ACSCs**						
Hospitalizations or ED visits for ACSCs per 1000 patient-days	0.14 (0.14-0.14)	0.27 (0.27-0.28)^a^	0.25 (0.24-0.25)	0.28 (0.26-0.31)	0.42 (0.40-0.44)	0.41 (0.39-0.43)
Mean total hospital LOS for ACSCs over 3 y, d	0.46 (0.45-0.47)	1.00 (0.90-1.10)^b^	0.92 (0.86-0.88)	1.00 (0.80-1.10)	1.10 (1.00-1.20)	1.70 (1.50-1.80)

Abbreviations: ACSC, ambulatory care–sensitive condition; ED, emergency department; LOS, length of stay.

^a^ Denotes *P* < .001 for comparison between mental health disorder absent and mental health disorder present based on *t* test.

^b^ Denotes *P* < .001 for comparison between mental health disorder absent and mental health disorder present based on *z* test.

^c^ Chronic disease defined as asthma, chronic heart failure, myocardial infarction, diabetes, epilepsy, hypertension, and chronic pulmonary disease (chronic kidney disease excluded because no *International Classification of Diseases *code used for definition) (eTable 1 in the Supplement).

The rates per 1000 patient days of being hospitalized or visiting the ED and the mean total length of stay per person varied by mental health disorder (Table 3). The unadjusted rate of overall ED visits per 1000 patient days was 1.75 (95% CI, 1.74-1.76; *P* < .001) for patients without a mental health disorder and 3.75 (95% CI, 3.72-3.79; *P* < .001) for those with a mental health disorder. The mean total hospital length of stay per person over 3 years was 4.7 (95% CI, 4.6-4.7; *P* < .001) days for those without a mental health disorder and 11.6 (95% CI, 11.4-11.8; *P* < .001) days for those with a mental health disorder. A similar trend was noted when restricting these health care visits specifically to chronic disease visits (Table 3).

Looking at the subset of hospital admissions, ED visits, and the mean total hospital length of stay per person for ACSCs, the rate of hospitalization or ED visits per 1000 patient days was 0.14 (95% CI, 0.14-0.14; *P* < .001) for those without a mental health disorder and 0.27 (95% CI, 0.27-0.28; *P* < .001) for those with a mental health disorder. The mean length of stay per person was 0.46 (95% CI, 0.45-0.47; *P* < .001) days for those without a mental health disorder and 1.0 (95% CI, 0.9-1.1; *P* < .001) days for those with a mental health disorder (Table 3).

### Costs Related to Care for ACSCs

Having a mental health disorder was associated with higher costs compared with not having a mental health disorder for hospitalization ($1290 [95% CI, $1230-$1340] vs $600 [95% CI, $590-$620]; *P* < .001) and for ED visits ($100 [95% CI, $98-$103] vs $56 [95% CI, $55-$57] *P* < .001). Overall, total costs for ACSCs were $1390 (95% CI, $1330-$1440; *P* < .001) for those with a mental health disorder and $660 (95% CI, $650-680; *P* < .001) for those without a mental health disorder (Table 4).

**Table 4.** Mean Total 3-Year Unadjusted Costs for Hospitalizations and ED Visits Associated With ACSCs for Adults With Chronic Disease, With and Without Mental Health Disorders

Resource	Mean Cost (95% CI), Can$^a^					
	No Mental Health Disorder Coded (n = 835 149)	Mental Health Disorder Coded (n = 156 296)	Depression (n = 110 671)	Schizophrenia (n = 13 320)	Alcohol Use Disorder (n = 40 955)	Drug Use Disorder (n = 31 465)
Hospitalizations	600 (590-620)	1290 (1230-1340)^b^	1170 (1110-1230)	1250 (1080-1410)	1940 (1820-2070)	1690 (1540-1830)
ED visits	56 (55-57)	100 (98-103)^b^	93 (90-95)	100 (98-103)	150 (143-155)	150 (148-155)
Total ACSC cost	660 (650-680)	1390 (1330-1440)^b^	1260 (1200-1330)	1350 (1180-1510)	2090 (1960-2230)	1830 (1690-1990)

Abbreviations: ACSCs, ambulatory care–sensitive condition; ED, emergency department.

^a^ All cost estimates are adjusted to 2016 Canadian dollars.

^b^ Denotes *P* < .001 for comparison between mental health disorder absent and mental health disorder present.

## Discussion

For Alberta residents with chronic disease, having a mental health disorder was associated with higher resource use and costs. The additional clinical and economic burden varied by mental health disorder, but higher costs were associated with age, male sex, low income, and increasing number of comorbidities. The trend was consistent even when mental health disorders were isolated from each other. Schizophrenia was associated with the highest total costs, while alcohol and drug use disorder were associated with the highest rates of hospital and ED visits overall and for ACSCs. Having depression was associated with lower costs compared with other mental health disorders; however, given its higher prevalence, the population impact and effect on health care budgets is substantial.

Previous studies have highlighted an association of mental health disorders with increased utilization and costs in individuals with chronic diseases,^17,18,19^ predominantly within individual chronic diseases and individual mental health disorders^20,36,37,38,39,40,41,42^ or within individual chronic diseases and multiple mental health disorders.^21,43,44,45,46,47^ A US report, which unlike this study did not use validated algorithms to define chronic diseases or mental health disorders (which increase diagnostic accuracy), showed that the presence of anxiety and depression increased costs by 33% to 169%.^17^ Aside from this US study, other published studies have been small and in selected cohorts, and most have not stratified by specific mental health disorders. Our study has highlighted differences in the association of specific mental health disorder with costs. This points to the possibility that different interventions may be required to improve care for people with different mental health disorders. Moreover, finding that alcohol and drug use disorders are associated with increased hospitalization and ED use for ACSCs is consistent with prior studies^48,49,50,51^ and offers insight into potential targets for intervention.

Our findings raise questions about optimizing the management of mental health disorders in patients with chronic disease. Higher costs and utilization among those with mental health disorders do not appear to be driven by acute care specifically for mental health disorders or chronic diseases. People with co-occurring chronic diseases and mental health disorders are a heterogenous group with a myriad of health and social needs, and the drivers of their acute care use are likely multifactorial, possibly including poor access to coordinated care and mental health care delivered by nonspecialists, among others. Studies have shown improved medical outcomes when treating depression.^52,53^ However, there are mixed results in the use of substance use screening and brief intervention in medical settings.^54,55,56,57,58,59,60,61,62,63^ Thus, additional research is needed to determine how best to optimize mental health among people with chronic disease.

Our results also raise the question of whether a different model of care for patients with mental health disorders and chronic disease needs to be developed. For example, integration of medical and psychiatric health care has been explored in primary care^64,65^ and specialty care,^66^ using mental health case managers^67^ and nurse practitioners.^68^ Although this is a developing area, studies have shown reductions in total costs estimated at between 5% and 16%, with evidence being most robust in elderly patients with depression.^18,69,70,71^ Other studies have shown a strong case for investing in mental health, with potential long-term cost savings despite upfront spending, among other benefits to overall wellness.^72,73,74,75^ Further, payment models could be developed to support or encourage new models of care, such as a bundled payment that includes the costs of chronic disease and mental health care services. While bundled payments for chronic diseases^76^ and mental health and substance use disorders^77^ can be challenging, bundled payment for diabetes care was found to improve care coordination,^78^ and we did identify variation in costs (eg, drug spending) that point to areas where savings under a bundled payment model may be plausible.^79^

### Strengths and Limitations

Strengths of our study include that we conducted a population-based analysis of all adults in Alberta with chronic disease, and while our results may not be applicable to all settings, we expect similar trends in other jurisdictions. We measured a comprehensive set of health care costs across a variety of mental health disorders, using validated definitions to reduce misclassification. To our knowledge, this is the first study analyzing the association of these individual mental health disorders with costs of care for adults with chronic disease.

This study has limitations. It was a retrospective cohort study using administrative health data, and thus, the limitations of using administrative health data, such as lack of detail regarding circumstances around health visits, apply to our study. We were also unable to control for variables such as severity of disease, but our regression model still identified a strong association of mental health disorders with costs after controlling for the covariates. Further, we are unable to comment on the disease trajectory and delineate whether severe physical chronic disease leads to development of mental health issues vs mental illness leading to higher costs through the development of chronic physical illness.

## Conclusions

We found that a co-occurring mental health disorder was strongly associated with an increased cost of care for patients with chronic diseases. The underlying reasons for this association cannot be determined given our limitations. Further research is needed on 2 fronts to inform clinicians and policy makers about areas where additional investment could improve health and reduce net health care costs. First, how the presence of a mental health disorder influences the course of a chronic disease needs to be clarified. Overlapping risk factors need to be identified, and the sequence of diagnoses could be considered. Second, studies should assess the design and impact of interventions aimed at improving mental health in people with chronic disease, including the potential for integrated medical and psychiatric care and bundled payments to improve outcomes and lower costs.

## Notes

Supplement. **eTable 1.** Administrative Data Codes Used to Define Chronic Diseases **eTable 2.** Administrative Data Codes Used to Define Mental Health Disorders **eTable 3.** Administrative Data Codes Used to Define Ambulatory Care–Sensitive Conditions **eTable 4.** Mean 3-Year Unadjusted Costs for Adults With Chronic Disease, With and Without a Mental Health Disorder, by Age, Sex, Income, and Rural Status **eTable 5.** Mean 3-Year Unadjusted Costs for Adults With Chronic Disease, With and Without a Mental Health Disorder, by Isolated Mental Health Disorder and Number of Comorbidities **eTable 6.** Parameter Estimates for Ordinary Least Squares Model for Total Costs **eFigure.** Co-occurrence of Depression, Schizophrenia, Alcohol Use Disorder, and Drug Use Disorders Among People With Chronic Disease and a Mental Health Disorder (N = 156 296) **eReferences.**
