# Supplementary material for: Association of Mental Health Disorders With Health Care Utilization and Costs Among Adults With Chronic Disease
Source: JAMA Netw Open. 2019 Aug 23;2(8):e199910. doi: 10.1001/jamanetworkopen.2019.9910 (PMC6714022; doi:10.1001/jamanetworkopen.2019.9910)
Supplement: Supplement. — eTable 1. Administrative Data Codes Used to Define Chronic Diseases eTable 2. Administrative Data Codes Used to Define Mental Health Disorders eTable 3. Administrative Data Codes Used to Define Ambulatory Care–Sensitive Conditions eTable 4. Mean 3-Year Unadjusted Costs for Adults With Chronic Disease, With and Without a Mental Health Disorder, by Age, Sex, Income, and Rural Status eTable 5. Mean 3-Year Unadjusted Costs for Adults With Chronic Disease, With and Without a Mental Health Disorder, by Isolated Mental Health Disorder and Number of Comorbidities eTable 6. Parameter Estimates for Ordinary Least Squares Model for Total Costs eFigure. Co-occurrence of Depression, Schizophrenia, Alcohol Use Disorder, and Drug Use Disorders Among People With Chronic Disease and a Mental Health Disorder (N = 156 296) eReferences. [file jamanetwopen-2-e199910-s001.pdf]

Supplementary Online Content

Sporinova B, Manns B, Tonelli M, et al. Association of mental health disorders with health care utilization and costs among adults with chronic disease. *JAMA Netw Open*. 2019;2(8):e199910. doi:10.1001/jamanetworkopen.2019.9910

eTable 1. Administrative Data Codes Used to Define Chronic Diseases
eTable 2. Administrative Data Codes Used to Define Mental Health Disorders
eTable 3. Administrative Data Codes Used to Define Ambulatory Care–Sensitive Conditions
eTable 4. Mean 3-Year Unadjusted Costs for Adults With Chronic Disease, With and Without a Mental Health Disorder, by Age, Sex, Income, and Rural Status
eTable 5. Mean 3-Year Unadjusted Costs for Adults With Chronic Disease, With and Without a Mental Health Disorder, by Isolated Mental Health Disorder and Number of Comorbidities
eTable 6. Parameter Estimates for Ordinary Least Squares Model for Total Costs
eFigure. Co-occurrence of Depression, Schizophrenia, Alcohol Use Disorder, and Drug Use Disorders Among People With Chronic Disease and a Mental Health Disorder (N=156,296)
eReferences.

This supplementary material has been provided by the authors to give readers additional information about their work.

© 2019 Sporinova B et al. *JAMA Network Open.*

eTable 1. Administrative Data Codes Used to Define Chronic Diseases

Chronic Disease	Diagnostic Algorithm	Sensitivity	PPV	ICD-9	ICD-10
**Asthma**	1 hospitalization or 3 ACCS in 2 years or less[1]	74% (ICD-9 CM)	72%	493	J45
**CHF**	1 hospitalization or 2 claims in 2 years or less[1]	91% (ICD-9 CM)<br><br>90% (ICD-10)	72% (ICD-9 CM)<br><br>69% (ICD-10)	398.91, 402.01, 402.11, 402.91, 404.01, 404.03, 404.11, 404.13, 404.91, 404.93, 425.4–425.9, 428	I09.9, I25.5, I42.0, I42.5–I42.9, I43, I50
**Myocardial Infarction**	1 hospitalization[1]	89% (ICD-9 CM)	89% (ICD-9 CM)	410	I21-I22
**Diabetes**	1 hospitalization or 2 claims in 2 years or less[1]	86% (ICD-9 CM)	80% (ICD-10)	250	E10-E14
**Epilepsy**	1 most responsible hospitalization or 2 claims in 2 years or less or 1 most responsible ACCS[1]	N/A (ICD-9 CM and ICD-10)	99% (ICD-9 CM and ICD-10)	345	G40-G41
**HTN**	1 hospitalization or 2 claims in 2 years or less[1]	79% (ICD-9 CM)<br>68% (ICD-10)	95% (ICD-9 CM)<br>93% (ICD-10)	401-405	I10-I13, I15
**Chronic pulmonary disease**	1 hospitalization or 2 claims in 2 years or less[1]	55% (ICD-9 CM)<br><br>53% (ICD-10)	92% (ICD-9 CM)<br><br>91% (ICD-10)	416.8, 416.9, 490–492, 494-505, 506.4, 508.1, 508.8	I27.8, I27.9, J40–J44, J46-J47, J60–J67, J68.4, J70.1, J70.3
**CKD**	Mean eGFR <60mL/min*1.73 m2 in 2 years or less; if no GFR measurement, ACR > or equal to 3mg/mmol ; if no ACR measure, PCR >= 15; if no PCR, UDIP >= 1+ (all prior 2 years)[2]	N/A	N/A	N/A	N/A

© 2019 Sporinova B et al. *JAMA Network Open.*

eTable 2. Administrative Data Codes Used to Define Mental Health Disorders

Mental Health Disorder	Diagnostic Algorithm	Sensitivity	PPV	ICD-9	ICD-10
**Depression**	1 hospital or 2 physician claims in 2 years or less[1]	57% (ICD-9-CM)<br><br>45% (ICD-10)	80% (ICD 9-CM)<br><br>92% (ICD-10)	296.2, 296.3, 300.4, 309, 311 (excluding 296.5 bipolar depression)	F20.4, F 31.3-31.5, F32, F33 (excluding 33.4x as MDD in remission), F34.1, F41.2, F43.2
**Schizophrenia**	1 hospital or 2 physician claims in 2 years or less[1]	87% (ICD-9-CM)	87% (ICD-9-CM)	295	F20, F21, F23.2, F25
**Alcohol use disorder**	1 hospital or 2 physician claims in 2 years[1]	54% (ICD-9-CM)<br><br>52% (ICD-10)	83% (ICD-9-CM)<br><br>84% (ICD-10)	265.2, 291.1- 291.3, 291.5-291.9, 303.0, 303.9, 305.0, 357.5, 425.5, 535.3, 571.0-571.3, 980, V11.3	E52,F10, G62.1, I42.6, K29.2, K70.0, K70.3, K70.9, T51, Z50.2, Z71.4, Z72.1
**Drug use disorder**	1 hospital or 2 physician claims in 2 years (algorithm created, ICD codes verified [3])	55.3% (ICD-9-CM)<br><br>46.7% (ICD-10)	73.7% (ICD-9-CM)<br><br>81.4% (ICD-10)	292.X, 304.X, 305.2-305.9, V65.42	F11.X-F16.X, F18.X, F19.X, Z71.5, Z72.2

© 2019 Sporinova B et al. *JAMA Network Open.*

eTable 3. Administrative Data Codes Used to Define Ambulatory Care–Sensitive Conditions

Outcome	ICD-9 codes	ICD-10 codes	Exclusions	Source
**Grand mal status and other epileptic convulsions**	345	G40, G41	none	CIHI[4]
**Chronic obstructive pulmonary disease (COPD)**	491, 492, 494, 496	J41, J42, J43, J44, J47	none	CIHI[4]
**Acute lower respiratory infection (MRDx)**	466, 480, 481, 482, 483, 485, 486, 487.0	J10.0, J11.0, J12, J13, J14, J15, J16, J18, J20, J21, J22	none	CIHI[4]
**Asthma**	493	J45		CIHI[4]
**Diabetes**	250.0, 250.1, 250.2, 250.7, 250.8	E10.0, E10.1, E10.63, E10.9, E11.0, E11.1, E11.63, E11.9, E13.0, E13.1, E13.63, E13.64, E13.9, E14.0, E14.1, E14.63, E14.64, E14.9	None	CIHI[4]
**Hypertension**	401.0, 401.9, 402.0, 402.1, 402.9	I10.0, I10.1, I11	None	CIHI[4]
**Heart Failure**	428, 518.4	I50, J81	None	CIHI[4]
**Angina**	411, 411.1, 411.8, 413	I20, I23.82, I24.0, I24.8, I24.9	See below	CIHI[4]
**Codes used to define ambulatory care sensitive conditions related to chronic kidney disease**	**ICD-9 codes**	**ICD-10 codes**	**Exclusions**	**Source**
**Diabetes w/ ketoacidosis**	250.1	E10.10, E10.12, E11.10, E11.12, E13.10, E13.12, E14.10, E14.12	None	Gao et al. [2]
**Diabetes w/ hyperosmolarity**	250.2	E10.00 + E87.0<br>E11.00 + E87.0	None	Gao et al. [2]
**Volume overload**	276.6	E87.7	None	Gao et al. [2]
**Hyperkalemia**	276.7	E87.5	None	Gao et al. [2]

© 2019 Sporinova B et al. *JAMA Network Open.*

Codes used to define ambulatory care sensitive conditions related to chronic kidney disease	ICD-9 codes	ICD-10 codes	Exclusions	Source
**Malignant hypertension**	401.0, 402.00, 402.01, 403.0, 404.0, 405.0	I10.1	None	Gao et al. [2]
**Heart failure**	428.x, 398.91, 402.01, 402.11, 402.91, 404.01, 404.03, 404.11, 404.13, 404.91, 404.93, 425.4, 425.5, 425.6, 425.7, 425.8, 425.9	I109.9, I25.5, I42.0, I42.5, I42.6, I42.7, I42.8, I42.9, I43.0, I43.1, I43.2, I43.8, I50.0, I50.1, I50.9	None	Gao et al. [2]

List of cardiac procedure codes for exclusion:
CCP: 47^^, 480^–483^, 489.1, 489.9, 492^–495^, 497^, 498^
ICD-9-CM: 336, 35^^, 36^^, 373^, 375^, 377^, 378^, 379.4–379.8
CCI: 1.HA.58.^^, 1.HA.80.^^, 1.HA.87.^^, 1.HB.53.^^, 1.HB.54.^^, 1.HB.55.^^, 1.HB.87.^^, 1.HD.53.^^, 1.HD.54.^^, 1.HD.55.^^, 1.HH.59.^^, 1.HH.71.^^, 1.HJ.76.^^, 1.HJ.82.^^, 1.HM.57.^^, 1.HM.78.^^, 1.HM.80.^^, 1.HN.71.^^, 1.HN.80.^^, 1.HN.87.^^, 1.HP.76.^^, 1.HP.78.^^, 1.HP.80.^^, 1.HP.82.^^, 1.HP.83.^^, 1.HP.87.^^, 1.HR.71.^^, 1.HR.80.^^, 1.HR.84.^^, 1.HR.87.^^, 1.HS.80.^^, 1.HS.90.^^, 1.HT.80.^^, 1.HT.89.^^, 1.HT.90.^^, 1.HU.80.^^, 1.HU.90.^^, 1.HV.80.^^, 1.HV.90.^^, 1.HW.78.^^, 1.HW.79.^^, 1.HX.71.^^, 1.HX.78.^^, 1.HX.79.^^, 1.HX.80.^^, 1.HX.83.^^, 1.HX.86.^^, 1.HX.87.^^, 1.HY.85.^^, 1.HZ.53 rubric (except 1.HZ.53.LA-KP), 1.HZ.54.^^, 1.HZ.55 rubric (except 1.HZ.55.LA-KP), 1.HZ.56.^^, 1.HZ.57.^^, 1.HZ.59.^^, 1.HZ.80.^^, 1.HZ.85.^^, 1.HZ.87.^^, 1.IF.83.^^, 1.IJ.50.^^, 1.IJ.54.GQ-AZ, 1.IJ.55.^^, 1.IJ.57.^^, 1.IJ.76.^^, 1.IJ.80.^^, 1.IJ.86.^^, 1.IK.50.^^, 1.IK.57.^^, 1.IK.80.^^, 1.IK.87.^^, 1.IN.84.^^, 1.LA.84.^^, 1.LC.84.^^, 1.LD.84.^^, 1.YY.54.LA-NJ, 1.YY.54.LA-FS, 1.YY.54.LA-NM

© 2019 Sporinova B et al. *JAMA Network Open.*

eTable 4. Mean 3-Year Unadjusted Costs for Adults With Chronic Disease, With and Without a Mental Health Disorder, by Age, Sex, Income, and Rural Status

Total cost, $ (95% CI*)	No Mental Health Disorder Coded	Depression only	Schizophrenia only	Alcohol Use Disorder only	Drug Use Disorder only	Depression + Alcohol Use Disorder	Depression + Drug Use Disorder	Depression + Alcohol Use Disorder + Drug Use	Alcohol Use Disorder + Drug Use	Schizophrenia + all other mental health disorders
**N**	835,149	89,293	4,791	21,068	10,848	5,483	5,448	4,445	6,401	8,529
**Overall**	20,120 (19,674 – 20,750)	29,830 (28,650-31,010)	34,259 (32,323 – 36,109)	34,984 (31,612 – 38,356)	35,356 (28,936 – 41,775)	48,379 (40,044 – 56,714)	48,409 (40,044 – 56,714)	55,656 (53,147 – 58,165)	37,959 (35,648 – 40,270)	59,544 (55,010 – 64,079)
**N**	303,122	27,987	1,583	7,019	2,212	1,427	913	419	690	1,558
**Age +65**	29,401 (28,577–30,227)	41,326[a] (40,016–42,636)	37,753 (34,688–40,818)	48,088[a] (39,838–56,338)	58,905[a] (31,040–86,770)	75,496[a] (44,134–106,857)	66,194[a] (60,604 – 71,783)	74,119[a] (65,415–82,823)	54,049[b] (47,377–60,720)	73,265[a] (50,808–95,722)
**N**	420,798	61,409	2,551	5,946	5,406	2,596	3,589	2,199	2,342	4,104
**Female**	19,656 (18,995 – 20,316)	29,122[a] (27,689 – 30,558)	34,048[a] (31,192 – 36,904)	32,444[a] (26,292 – 38,596)	37,433[a] (25,956 – 48,012)	42,663[a] (39,936 – 45,390)	46,694[a] (44,110 – 49,278)	56,329[a] (52,774 – 59,883)	35,795[a] (32,434 – 39,155)	64,116[a] (55,178 – 73,064)
**N**	200,129	22,362	1,730	6,542	3,485	1,678	1,835	1,777	2,381	3,353
**Low Income**	22,397 (21,230 – 23,563)	32,837[a] (29,523 – 36,150)	36,762[c] (33,576 – 39,947)	34,957[a] (29,840 – 40,073)	36,829[b] (29,536 – 44,121)	56,804[a] (30,086 – 83,523)	46,977[a] (43,408 – 50,546)	57,496[a] (53,720 – 61,273)	42,283[a] (38,343 – 46,223)	64,154[a] (53,520 – 74,790)
**N**	117,362	10,314	456	4,392	1,680	800	716	692	1,266	833
**Rural**	23,396 (21,635 – 25,157)	32,785[b] (27,460 – 38,110)	31,926 (21,989 – 41,863)	37,466[b] (28,646 – 46,287)	38,222[b] (23,651 – 52,793)	40,317 (35,417 – 45217)	46,207[c] (40,499 – 51,915)	51,573[c] (45,475 – 57,671)	37,540 (33,046 – 42,034)	52,610[b] (45,972 – 59,249)

*CI = Confidence interval; [a] Denotes a significant p-value <0.001 between mental health disorder absent and mental health disorder present; [b]Denotes p<0.01; [c]Denotes p<0.05; All cost estimates are adjusted to 2016 Canadian dollars

© 2019 Sporinova B et al. *JAMA Network Open.*

eTable 5. Mean 3-Year Unadjusted Costs for Adults With Chronic Disease, With and Without a Mental Health Disorder, by Isolated Mental Health Disorder and Number of Comorbidities

Total cost, $ (95% CI*)	No Mental Health Disorder Coded	Depression only	Schizophrenia only	Alcohol Use Disorder only	Drug Use Disorder only	Depression + Alcohol Use Disorder	Depression + Drug Use Disorder	Depression + Alcohol Use Disorder + Drug Use Disorder	Alcohol Use Disorder + Drug Use Disorder	Schizophrenia + all other mental health disorders
**N**	835,149	89,293	4,791	21,068	10,848	5,483	5,448	4,445	6,401	8,529
**Overall**	20,120 (19,674 – 20,750)	29,830 (28,650-31,010)	34,259 (32,323 – 36,109)	34,984 (31,612 – 38,356)	35,356 (28,936 – 41,775)	48,379 (40,044 – 56,714)	48,409 (40,044 – 56,714)	55,656 (53,147 – 58,165)	37,959 (35,648 – 40,270)	59,544 (55,010 – 64,079)
**N**	312,768	24,403	1,193	4,942	2,780	1,110	1,016	861	1,566	1,945
**1 comorbidity**	10,700 (10,125 – 11,276)	15,615[a] (14,009 – 17,221)	25,171[b] (20,885 – 29,457)	21,048[a] (13,796 – 28,300)	15,149 (12,912 – 17,385)	22,656[c] (20,286 – 25,026)	25,744[b] (21,224 – 30,264)	30,942[a] (26,957 – 34,927)	18,027 (15,820 – 20,234)	45,439[a] (40,037 – 50,841)
**N**	243,863	23,847	1,222	5,307	2,893	1,289	1,227	1,011	1,665	2,006
**2 comorbidities**	16,483 (15,537 – 17,429)	23,521[a] (20,409 – 26,635)	29,990[b] (26,920 – 33,059)	21,688 (20,070 – 23,305)	20,387 (18,506 – 22,267)	30,747[c] (28,248 – 33,245)	37,924[b] (23,310 – 52,538)	41,681[a] (37,682 – 45,679)	27,049 (24,015 – 30,082)	47,103[a] (43,824 – 50,383)
**N**	259,927	36,231	2,123	9,567	4,488	2,615	2,682	2,241	2,817	3,912
**3-6 comorbidities**	32,069 (30,826 – 33,311)	38,102[a] (36,497 – 39,708)	39,362 (36,472 – 42,252)	42,036[b] (37,878 – 46,194)	51,659[a] (36,313 – 67,005)	61,266[a] (43,985 – 78,547)	52,658[a] (49,461 – 55,855)	63,616[a] (59,879 – 67,352)	48,972[b] (44,574 – 53,370)	67,513[a] (58,300 – 76,726)
**N**	18,591	4,802	253	1,252	687	469	513	332	353	666

© 2019 Sporinova B et al. *JAMA Network Open.*

**>6 comorbidities**	63,304 (57,905 – 68,703)	71,044 (66,000 – 76,087)	74,915[c] (64,285 – 85,546)	92,463 (55,973 – 128,964)	73,656 (66,439 – 80,873)	85,858 (75,807 – 95,910)	96,080[c] (85,982 – 106,178)	108,580[c] (95,238 – 121,911)	89,950 (77,259 – 102,642)	91,400 (82,177 – 100,630)

*CI = Confidence interval; [a] Denotes a significant p-value <0.001 between mental health disorder absent and mental health disorder present; [b]Denotes p<0.01; [c]Denotes p<0.05; All cost estimates are adjusted to 2016 Canadian dollars

© 2019 Sporinova B et al. *JAMA Network Open.*

eTable 6. Parameter Estimates for OLS Model for Total Costs

Variable	Coefficient	P-value
**Constant**	$-17,025	0.000
**Patient Characteristics**		
**Low income**	$1,929	0.001
**Rural**	$2,465	0.001
**Age**	$562	0.000
**Age^2**	$-3	0.000
**Female**	$-1,379	0.007
**Mental Health Conditions**		
**Depression**	$8,117	0.000
**Schizophrenia**	$17,179	0.000
**Alcohol use disorder**	$7,690	0.000
**Drug use disorder**	$11,996	0.000
**Comorbidities**		
**Asthma**	$8,392	0.000
**Atrial fibrillation**	$8,084	0.000
**Lymphoma**	$47,664	0.000
**Cancer - metastatic**	$36,701	0.000
**Cancer – nonmetastatic**	$15,270	0.000
**Congestive heart failure**	$10,470	0.000
**Chronic kidney disease**	$11,393	0.000
**Chronic pain**	$6,507	0.000
**Chronic obstructive lung**	$8,313	0.000
**Cirrhosis**	$35,673	0.000
**Dementia**	$-3,527	0.018
**Diabetes**	$7,145	0.000
**Epilepsy**	$16,615	0.000
**Hypertension**	$5,970	0.000
**Inflammatory bowel disease**	$18,203	0.000
**Multiple sclerosis**	$9,263	0.001
**Myocardial infarction**	$3,058	0.014
**Peptic ulcer disease**	$30,507	0.000
**Peripheral vascular disease**	$19,032	0.000
**Rheumatoid arthritis**	$8,993	0.000
**Stroke**	$5,707	0.000
**R-squared**	0.047	
**Number of observations**	991,445	
**Only significant predictors are presented in the table. The model also considered high income, hepatitis b, hypothyroidism, irritable bowel syndrome, Parkinson's disease, and psoriasis**		

© 2019 Sporinova B et al. *JAMA Network Open.*

eFigure 1. Co-occurrence of Depression, Schizophrenia, Alcohol Use Disorder, and Drug Use Disorders Among People With Chronic Disease and a Mental Health Disorder (N=156,296)

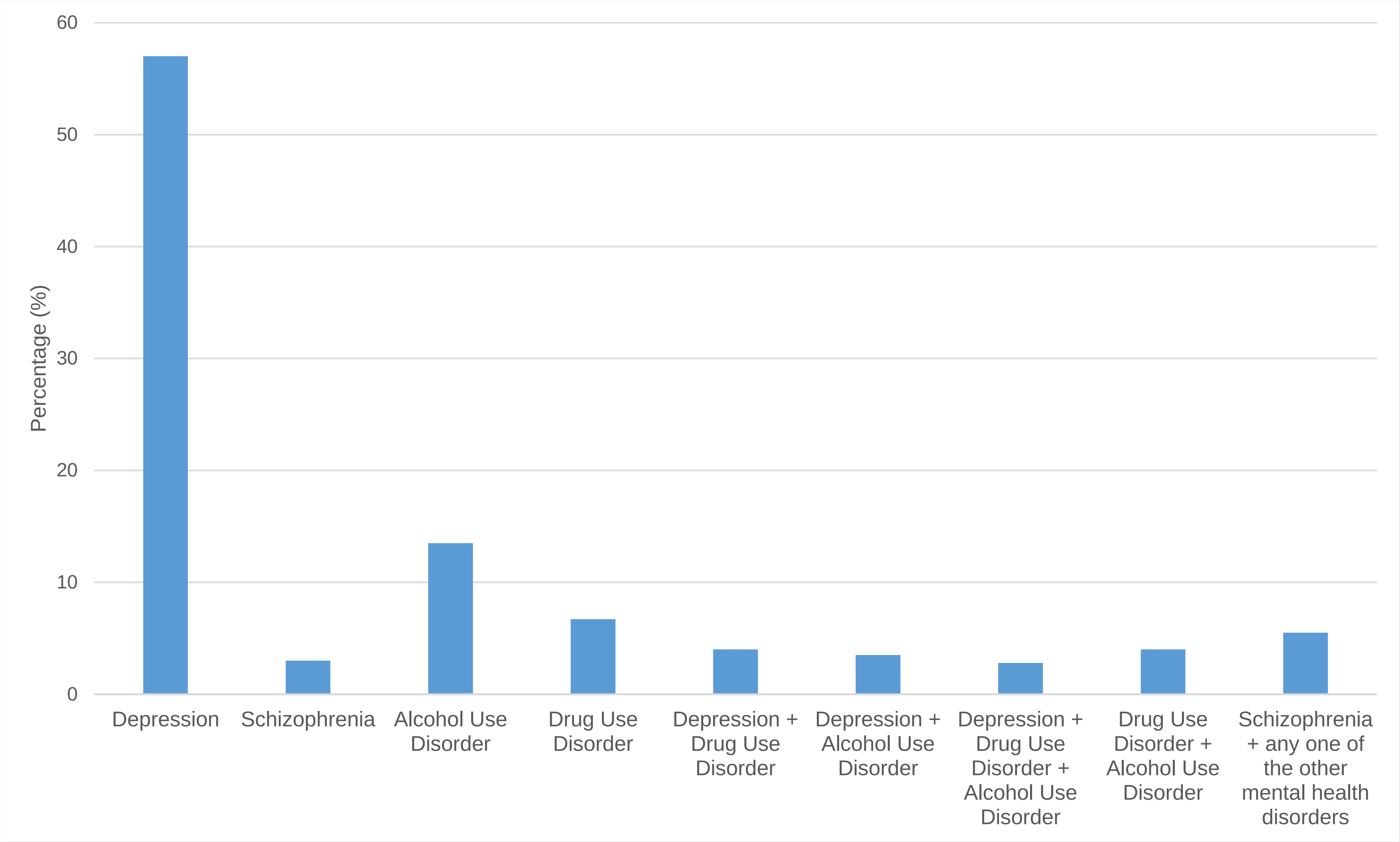

© 2019 Sporinova B et al. *JAMA Network Open.*

eReferences

1. Tonelli M, Wiebe N, Fortin M, et al. Methods for identifying 30 chronic conditions: application to administrative data. *BMC Med Inform Decis Mak.* 2015;15:31.
2. Gao S, Manns BJ, Culleton BF, et al. Access to health care among status Aboriginal people with chronic kidney disease. *CMAJ.* 2008;179(10):1007-1012.
3. Quan H, Li B, Saunders LD, et al. Assessing validity of ICD-9-CM and ICD-10 administrative data in recording clinical conditions in a unique dually coded database. *Health Serv Res.* 2008;43(4):1424-1441.
4. Canadian Institute for Health Information. Technical note: ambulatory care sensitive conditions (ACSC). 2010(May 27, 2018). www.cihi.ca/CIHI-ext-portal/pdf/internet/DEFINITIONS_052010_EN. Accessed April 7, 2017.

© 2019 Sporinova B et al. *JAMA Network Open.*
